# Marked Object Recognition Multitouch Screen Printed Touchpad for Interactive Applications

**DOI:** 10.3390/s17122786

**Published:** 2017-12-01

**Authors:** Jivago Serrado Nunes, Nelson Castro, Sergio Gonçalves, Nélson Pereira, Vitor Correia, Senentxu Lanceros-Mendez

**Affiliations:** 1 Center of Physics, University of Minho, 4710-057 Braga, Portugal; jivagonunes@gmail.com (J.S.N.); sgoncalves@engagelab.org (S.G.); nmmsp.18@gmail.com (N.P.); vcorreia@dei.uminho.pt (V.C.); 2 Centro ALGORITMI, University of Minho, Campus de Azurém, 4800-058 Guimarães, Portugal; 3 Basque Center for Materials, Applications and Nanostructures (BCMaterials), 48160 Derio, Spain; nelson.castro@bcmaterials.net; 4 engageLab, University of Minho, 4810-453 Guimarães, Portugal; 5 Ikerbasque, Basque Foundation for Science, Maria Diaz de Haro 3, 48013 Bilbao, Spain

**Keywords:** flexible sensors, touch sensors, object recognition, printable sensors, tactile data processing

## Abstract

The market for interactive platforms is rapidly growing, and touchscreens have been incorporated in an increasing number of devices. Thus, the area of smart objects and devices is strongly increasing by adding interactive touch and multimedia content, leading to new uses and capabilities. In this work, a flexible screen printed sensor matrix is fabricated based on silver ink in a polyethylene terephthalate (PET) substrate. Diamond shaped capacitive electrodes coupled with conventional capacitive reading electronics enables fabrication of a highly functional capacitive touchpad, and also allows for the identification of marked objects. For the latter, the capacitive signatures are identified by intersecting points and distances between them. Thus, this work demonstrates the applicability of a low cost method using royalty-free geometries and technologies for the development of flexible multitouch touchpads for the implementation of interactive and object recognition applications.

## 1. Introduction

Innovations in the areas of materials development and production are strongly affecting information and communication technologies, with a strong impact in a large variety of human activities, ranging from industrial processes to entertainment. Ubiquitous computation paradigms, tangible user interfaces, and physical computation have been playing an increasing role in the human–computer interaction field, giving rise to a new perspective of the fusion between computer and materials [1].

Circuit printing methods and sensitive electronics are enabling materials and digital technology to work together as complementing parts of novel devices, allowing to present information in a large variety of forms, colours, and textures [2,3,4] and at the same time allowing for the development of a new generation of sensors and actuators.

In this context, the touch sensors market and applications have experienced a strong growth mostly based on capacitive technologies, since the iPhone^®^ had its debut back in 2007 [5,6].

The projected capacitive technology can be implemented through two reading methods: the self-capacitance method and the mutual-capacitance. The self-capacitance consists in analyzing the capacitance of the electrodes to ground, when a finger is placed close together to the electrode. This allows the detection of just two fingers, though the hardware is of lower cost. On the other hand, the mutual-capacitance is a more robust method based on the mutual capacitance between two electrodes, where one electrode acts as a driver and the other one acts as a sensor. This method allows the detection of several touches, though it implies higher hardware costs. In both methods, the matrix of electrodes is scanned at high frequency [6,7]. Capacitive touch technology and market share is covered quite well in the literature [6,7].

Capacitive surfaces are composed by a dielectric substrate such as glass, acrylic, or polyester, and a coating based on a conductive transparent material. Indium tin oxide (ITO) conductive coating is the most used material in order to achieve the required conductivity and transparency, but the ITO deposition technology is not cost efficient [8,9], paving the way for replacement by printed technology solutions.

Together with transparent capacitive touch surfaces for big monitors, smartphones, and tablets, there is an increasing demand of opaque capacitive applications as well, such as keyboards [10], touchpads [11], position tracking surfaces [12,13], proximity buttons [14], and pen-shaped haptic devices [15].

Other applications that do not require transparency, such as humidity sensors, can also use capacitance variations to measure physical variables. These sensors can be produced by printing conductive ink over a polymer substrate [16,17].

The scientific and technological communities are addressing polymer-based sensors and sensor matrices for an increasing number of applications, as they are characterized by a cost-effective production based on additive manufacturing, being able to be produced on large scale [18,19]. Presenting further advantages, printing electronic components on plastic substrates offers devices the potential of being thin, foldable/rollable, lightweight, and wearable [20]. Thus, there is a growing trend on the development of polymers and polymer composites for the development of new devices [21,22,23].

In this work, we present a method for producing an opaque projected capacitive touch surface, based on the mutual-capacitance method, with the corresponding readout electronics and software data treatment. Using a screen printing method and silver ink, a Melinex^®^ substrate, and an adhesive PET encapsulation, a functional capacitive surface was fabricated. This surface also incorporates an object recognition module and multi-functional touch input for smart books or smart surface applications, among others. These functionalities enhance multimedia interaction content for development purposes and demonstrate the important possibilities offered by polymer-based materials for a new generation of low-cost capacitive touchscreens.

## 2. Materials and Methods

### 2.1. Printed Geometry

**Figure 1:**
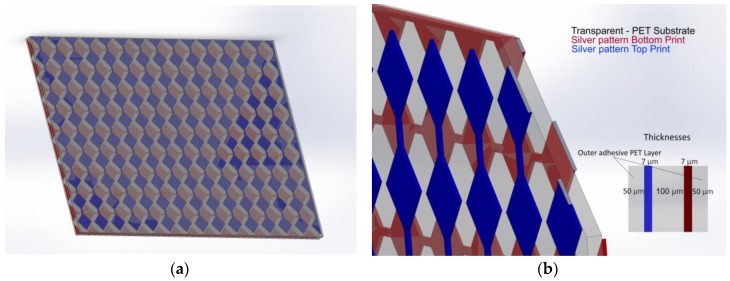
Illustration of the capacitive matrix silver electrodes geometry: (**a**) diamond electrodes pattern front view; (**b**) diamond electrodes pattern perspective view.

Capacitive sensors may have many different geometries, each with advantages and disadvantages as it is explored in [24]. In the present work, the selected geometry is based on a diamond pattern, which is one of the most commonly used. In such a pattern, the capacitors are spread over two layers and arranged in a diamond-like structure [9,25,26]. The dielectric material is between rows and columns, thus providing a capacitance between each intersection, providing the possibility to determine the touch location when the capacitance changes with the approximation of any surface with enough conductivity, such as skin. Figure 1 shows the geometries used for the development of the touchpad with an area of 102 × 67 mm^2^, and the corresponding layers.

Every capacitive square in the electrodes has an area of 16 mm^2^ except on the borders, which are 8 mm^2^ triangles, and the corners with an area of 4 mm^2^. With this resolution, it is possible to detect human fingertip size conductive objects. A similar size matrix with higher resolution would require a capacitive controller with a higher number of channels than the one used in the present work. Lowering the resolution could still be suitable to detect larger conductive areas like hands or feet. For non-transparent printed touchpads, conductive silver ink is a suitable choice, as it can be simply cured in a normal oven at a constant temperature, without temperature curve requirements or special treatments. In this work, the selected silver ink was HPS-21LV from Novacentrix^®^ (Austin, TX, USA), which was cured at 120 °C in a P Selecta oven model 2005165 for 30 min. The used substrate was Melinex^®^ (Lohmann Technologies UK Ltd., Milton Keynes, UK), which is a PET sheet with an ink adhesion treatment for improved printing process.

### 2.2. Capacitive Detection Circuit

**Figure 2:**
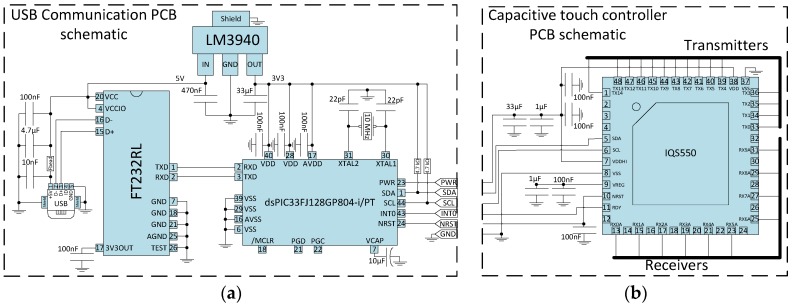
Schematic representation of the hardware: Communication PCB as I^2^C-USB converter (**a**) and capacitive controller instrumentation circuit (**b**).

The capacitive variation between the intersections on the matrix can be detected by amplitude [27,28] or frequency [29,30] analysis, methods that can be used to detect single point-to-point variations. However, for a matrix of capacitive sensors, a multiplexing method for rows and columns would be needed in order to sample each intersection. Using a commercial controller, this requirement is bypassed with the high number of reading channels. The capacitive controller used was the IQS550 from Azoteq Ltd. (Paarl, South Africa). According to manufacturer instructions, the complete interface circuit was designed as presented in Figure 2b, to obtain the touch locations of the trackpad. The digital data provided by the controller is sent through a firmware communication via I^2^C (Inter-Integrated Circuit) to the microcontroller dsPIC33FJ128GP804, from Microchip Technology Inc. (Chandler, ZA, USA).

In order to transmit the data from the IQS550, another Printed Circuit Board (PCB) was built to serve as interface to a USB terminal (Figure 2a). For a direct USB communication, a FT232RL from Future Technology Devices International Ltd. (Glasgow, UK) was used, trading data with the microcontroller through a UART (Universal Asynchronous Receiver/Transmitter) connection. 

### 2.3. Touchpad Fabrication

**Figure 3:**
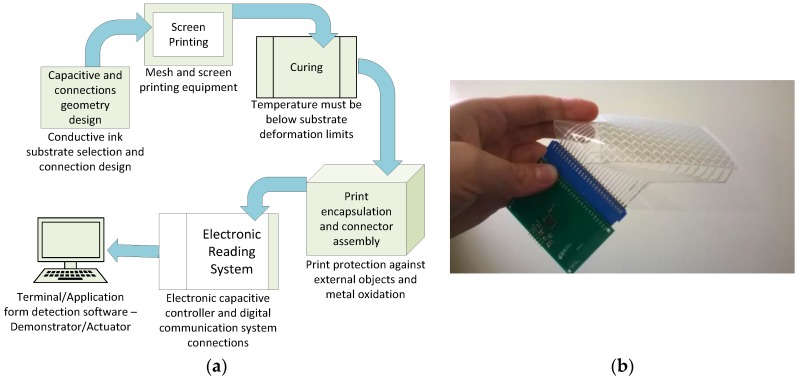
Process fabrication flow diagram (**a**) and printed capacitive matrix connected to the PCB with the capacitive touch controller IC (**b**).

The printing method was performed by screen printing with a polyester mesh with 62/64 wires. First, the matrix was printed in one side of the polymer (top print) and dried out at room temperature for 6 h. Then, the bottom side of the polymer was printed with the aid of aligning points. The completed matrix was cured then for 30 min at 120 °C in order to evaporate the solvent. The top layer print was not immediately cured in order to avoid mechanical shrinkage or deformation of the substrate, which would highly affect the alignment when printing the bottom layer pattern. Further, an Amphenol FCI connector was used for the electrical connection, and an adhesive PET layer was applied on each side of the film to protect the circuits from corrosion and oxidation, as well as to serve as an isolating layer between the printed circuit and the fingers. The FCI connector to the printed matrix contacts were fabricated by pressure sealing. The connectors perforate the PET substrate and keep a tight connection between the flexible silver traces and rigid tin. The complete system process is summarized in the diagram of the Figure 3a. The matrix was then connected to the capacitive controller board (schematic representation in Figure 2b). The final printed touchpad connected to the PCB is shown in Figure 3b. It is worth noting that the final surface is a ~220 µm thick plastic sheet, so it is a flexible structure to fit in any rigid plane or rounded surface.

## 3. Results and Discussion

The system performance was first evaluated in the application provided by Azoteq with a CT210 programmer. Thus, it was possible to obtain all the applied signal delays in each intersection and calibrate the sensitivity, approximation threshold, and balance of the capacitive intersections. A header file is generated in order to apply at the IQS550 at system start. The matrix calibration was achieved through a process of trial and error in order to get touch stability and precision. 

**Figure 4:**
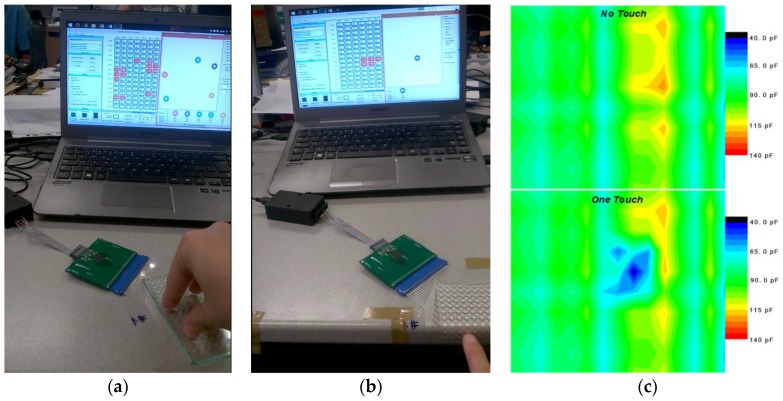
Test were performed with the CT210 programmer for the maximum of possible touches using a 5 mm thick glass as an overlay (**a**), folded surface finger detection (**b**) and capacitive distribution with glass overlay of the matrix with and without touch (**c**).

The surface displayed a suitable response to finger touch, though it was greatly improved with a glass overlay when fixed to a table in a flat disposition (Figure 4a). Removing the glass and folding the surface while fixing to the edge of a table still allowed the detection of the finger touch, demonstrating the flexibility of the touchpad (Figure 4b). Applying a covering glass as an extra overlay gives the system improved mechanical and electrical stability, as fingers cannot reach too close to the capacitors’ dielectric film and will not saturate the sensors as easily. It will also provide more steadiness to the contacts, as the terminals are the most sensitive part of the system in terms of mechanical stability. It worth noting that the use of a glass overlay requires a new software calibration, as the overall physical properties of the sensors are also changed. Any vibration, stretch, or deviation in the contact position can lead to variations of the electrical resistance of the rows and columns, affecting the signal response and unbalancing the capacitances along the matrix. Therefore, special attention must be paid to these connections. Figure 4c shows the capacitance distribution of the matrix with glass overlay with and without touch, based on the injected signal and circuit electrical resistance, revealing a fair distribution among the most part of the 150 channels with small deviations that are handled by the calibration software. The response time is 100 Hz for the 150 channels, though it decreases with every extra touch (a maximum of 5 going as low as 88 Hz). A fair capacitance drift of 40 pF between the extremes of the spectrum is observed, easily calibrated by software. A 30~50 pF variation can be found in the strongest points of the touch, providing good sensitivity for the controller to detect.

A C# application was developed in order to acquire the touch coordinates, using more than one touch position, such as touch identification and touch intensity. The IQS550 provided with 10 rows × 15 columns, a resolution of 2304 × 3584 points, converting into ~36 points/mm, allowing high precision and even the interpretation of the touch intensity, which is measured by the capacitive area triggered by finger expansion, as strength pressure (see Supplementary Materials). As for the reading process, the IC controller applies a high frequency signal in each column while grounding all the other columns (isolating a particular group of points between that column and all the rows simultaneously), then it grounds all other rows except one and measures the signal frequency and checks for delay. The method continues with the next row until the last, and then restarts with another column. The scanning frequency is 100 Hz.

**Figure 5:**
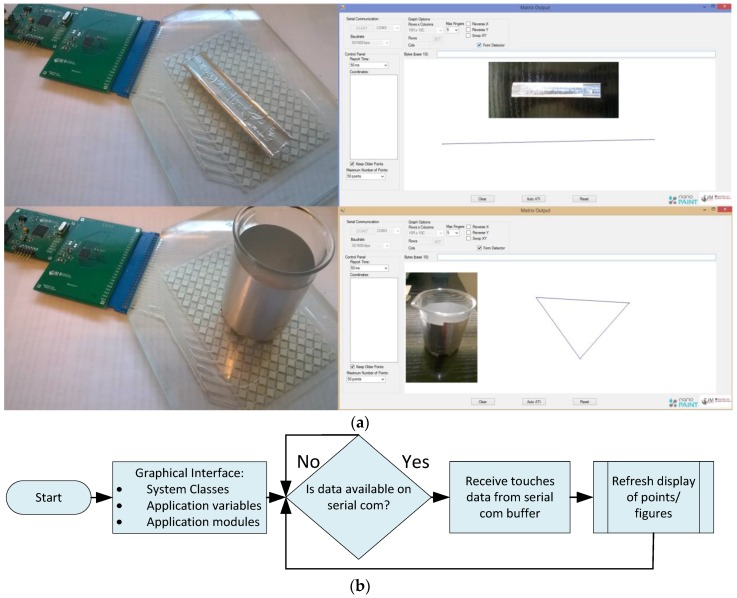
(**a**) Marked objects can be detected by software with the aid of a specific number of points and distances between then in a C# environment; (**b**) System process and software processing main algorithm.

Together with the readout and display software, a module was developed to identify marked objects with the aid of a specific number of points and distance between them that acted as a conductive signature for each object. Thus, a different pattern identified each object, allowing its recognition when in contact with the touchpad. The objects were marked using silver tape as a conductive material with small elevations (fingertip size) in order for the capacitive controller to identify each touching place (Figure 5a). 

The application is comprised of a graphical interface in order to select, configure, and connect to the serial COM (communication port) port in order to stream the data of the touches. The system control process is represented in Figure 5b. It is shown that the system can configure the display format, touch drawing refresh rate, number of displayed points, and enable object detection in relation to the different patterns to be recognized, among others.

Two different objects were used as demonstrator (see Supplementary Materials): a cup silver-coated with tape, a base of three points linked as a triangle, and a flat object with two identification points. Touching in the conductive tape of the object closes the circuit to the ground through the human body, allowing the objects to be identifiable through the touching points, as the distances between points fall within a certain range of tolerance, with an error of ~7%. This method is implemented with real-time calculations, as in [13] and it is valid for 2D objects. It is worth noting that the main issue is not the identification of the objects by the touchpad, but to identify the different shapes (patch patterns) by the software. 

These results demonstrate the versatility of the printed technologies in the replacement of the traditional rigid and flat interfaces with the user. Furthermore, as long as it is possible to keep contacts well fixed to the rigid parts, there is no commitment with a regular plane surface in a device. This means that we can place the touch panel on a curved surface, but after that it cannot be moved without recalibration to another type of surface.

## 4. Conclusions

The present work reports on the fabrication and performance of a touch and object detection printed capacitive matrix. The printed matrix was fabricated with a PET substrate and two printed silver layer patterns, one on each side. Adhesive PET was applied on both sides over the printed layers for protection against touches and oxidation. A commercial capacitive controller IC was used in order to convert the capacitance in each intersection to digital values, followed by communication to a microcontroller, which treats the data to be sent to a computer software application. The resolution of 36 points/mm obtained from the digital controller connected to the printed matrix enables handwriting applications, touch buttons, gesture detection, and hovering direction sensors. The inexpensive nature of the materials/quantities required allows the fabrication of larger surfaces (as large as the printed pattern electrical resistance allows). However, it is important to pay attention to the electrical noise introduced by mechanical deformations in the PCB connector through proximity, which can be greatly improved by using flexible ribbon cable connectors. 

Printing enables the application in any object and any plain or curved surface, such as in flexible paper sheets of a smart book, curved control touch panels in machines, robots or tubes to control flow/pressure, among others. Finally, its use for object interaction by capacitive pattern recognition has been demonstrated, relying on the number of points and distance between them with an error of ~7%. A good alignment between top and bottom patterns is required to obtain suitable response times and precision. The number of potential object signatures will strongly increase with surface size and number of points, allowing rich multimedia environments. Thus, this work demonstrates the suitability of printed technologies and polymer-based material for developing low-cost smart objects/surfaces for flexible multimedia applications.

## Supplementary Materials

The following are available online at http://www.mdpi.com/1424-8220/17/12/2786/s1.
